# MicroRNA-126b-5p Exacerbates Development of Adipose Tissue and Diet-Induced Obesity

**DOI:** 10.3390/ijms221910261

**Published:** 2021-09-23

**Authors:** Linyuan Shen, Jin He, Ye Zhao, Lili Niu, Lei Chen, Guoqing Tang, Yanzhi Jiang, Xiaoxia Hao, Lin Bai, Xuewei Li, Shunhua Zhang, Li Zhu

**Affiliations:** 1 College of Animal Science and Technology, Sichuan Agricultural University, Chengdu 611130, China; shenlinyuan0815@163.com (L.S.); hejin19960812@163.com (J.H.); zhye3@foxmail.com (Y.Z.); dky9829@126.com (L.N.); chenlei815918@163.com (L.C.); tyq003@163.com (G.T.); xiaoxia6363@126.com (X.H.); blin16@126.com (L.B.); lixuewei9125@126.com (X.L.); 2 Farm Animal Genetic Resources Exploration and Innovation Key Laboratory of Sichuan Province, Sichuan Agricultural University, Chengdu 611130, China; 3 College of Life Science, Sichuan Agricultural University, Chengdu 611130, China; jiangyz04@163.com

**Keywords:** miR-126b-5p, adipogenesis, fatty acid metabolism, insulin resistance

## Abstract

Obesity has become a worldwide epidemic, caused by many factors such as genetic regulatory elements, unhealthy diet, and lack of exercise. MicroRNAs (miRNAs) are non-coding single-stranded RNA classes, which are about 22 nucleotides in length and highly conserved among species. In the last decade, a series of miRNAs were identified as therapeutic targets for obesity. In the present study, we found that miR-126b-5p was associated with adipogenesis. miR-126b-5p overexpression promoted the proliferation of 3T3-L1 preadipocytes by upregulating the expression of proliferation-related genes and downregulating the expression of apoptosis-related genes; the inhibition of miR-126b-5p gave rise to opposite results. Similarly, miR-126b-5p overexpression could promote the differentiation of 3T3-L1 preadipocytes by increasing the expression of lipid deposition genes and triglyceride (TG) and total cholesterol (TC) levels. Moreover, luciferase reporter assay demonstrated that adiponectin receptor 2 (Adipor2) and acyl-CoA dehydrogenase, long chain (ACADL) were the direct target genes of miR-126b-5p. Moreover, overexpression of miR-126b-5p could exacerbate the clinical symptoms of obesity when mice were induced by a high-fat diet, including increased adipose tissue weight, adipocyte volume, and insulin resistance. Interestingly, overexpression of miR-126b-5p in preadipocytes and mice could significantly increase total fatty acid content and change the fatty acid composition of adipose tissue. Taken together, the present study showed that miR-126b-5p promotes lipid deposition in vivo and in vitro, indicating that miR-126b-5p is a potential target for treating obesity.

## 1. Introduction

As the quality of life continues to improve, overweight and obesity are becoming a noticeable trend in most countries. Obesity causes a series of diseases such as cardiovascular disease, chronic inflammation, non-alcoholic fatty liver disease, type 2 diabetes mellitus (T2DM), and even cancer [1,2,3,4]. A survey in 2017 showed that the prevalence of overweight was 33.8% (body mass index, BMI ≥ 24.0 kg/m^2^, according to Chinese criteria), and the obesity prevalence was 6.3% (BMI ≥ 28.0 kg/m^2^) among 15.8 million men in Chinese rural areas [5]. Other studies showed that global obesity prevalence would reach 18% in men and surpass 21% in women by 2025 if obesity is not controlled [6]. Therefore, obesity or overweight has become a global public health problem that cannot be ignored and should be dealt with urgently.

MicroRNAs (miRNAs) are a class of non-coding single-stranded RNA, about 22 nucleotides in length and highly conserved among species. miRNAs bind to the 3′ untranslated region (3′UTR) of target genes, disrupting their mRNA inactivation or transcription. Recently, more and more miRNAs associated with obesity or adipose tissue have been studied. For instance, Zhang et al. found that miR-143a-3p could inhibit preadipocyte proliferation and enhance their differentiation by targeting *MAPK7* [7]. Fan et al. reported that miR-152 had the same effect on 3T3-L1 preadipocytes; however, the difference was that the target gene was lipoprotein lipase (*LPL*) [8]. Similarly, Peng et al. found that miR-429 inhibited porcine preadipocyte differentiation and promoted their proliferation by targeting *KLF9* and *p27*, which promote preadipocyte differentiation and inhibit the cell cycle during cell proliferation, respectively [9]. Therefore, miRNAs play an essential role in the process of adipogenesis. Our previous study showed that the expression level of miR-126-5p in obese pigs was higher than that in lean pigs, which also positively correlated with the volume of adipocytes in several adipose tissues [10]. Therefore, we speculated miR-126-5p was a crucial regulatory factor in adipogenesis. 

However, all the previous studies on miR-126-5p have mainly focused on the development of diseases, such as cancer, atherosclerosis, myocardial infarction, T2DM, and angiogenesis [11,12,13,14]. However, its mechanism of regulating adipogenesis and obesity is still unclear. Therefore, in the present study, 3T3-L1 preadipocytes and C57BL/6 mice were used as models to investigate the effects of miR-126b-5p on adipogenesis in vitro and in vivo.

## 2. Results

### 2.1. miR-126b-5p Was Associated with Adipogenesis

**Figure 1:**
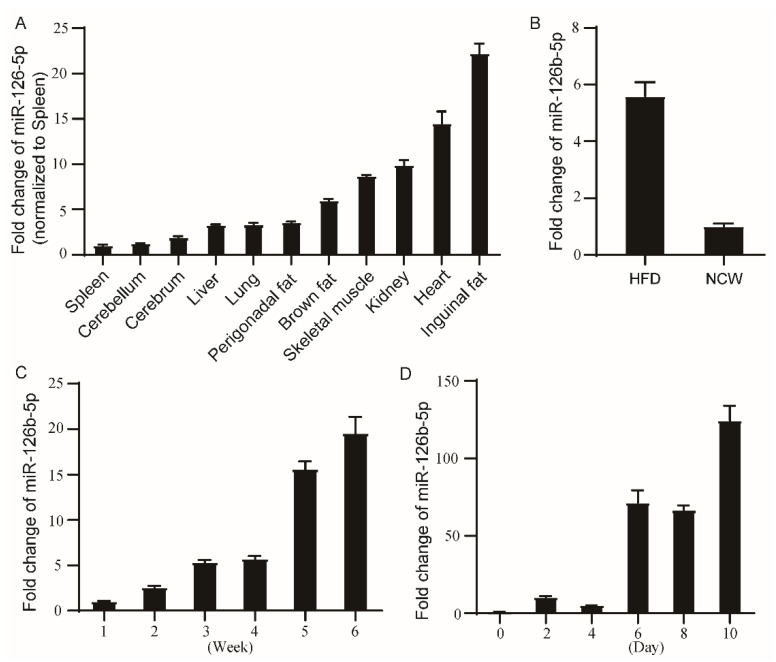
miR-126b-5p was positively correlated with adipogenesis. The expression of miR-126b-5p in different tissues of 8-week-old mice (**A**), inguinal adipose tissue of diet-induced obese mice (HFD) and normal mice (NCW) (**B**), inguinal adipose tissue of 1–6-week-old mice (**C**), and the differentiation stage of 3T3-L1 preadipocytes (**D**). The data are presented as mean ± SEM (*n* = 3).

To investigate the potential mechanism of miR-126b-5p in adipogenesis, we first determined whether the expression of miR-126b-5p was related to adipogenesis. As shown in Figure 1A, miR-126b-5p was expressed in different murine tissues, and the expression of miR-126b-5p was the highest in the inguinal adipose tissue compared with other tissues. Subsequently, the expression of miR-126b-5p was detected in inguinal adipose tissue of diet-induced obese (DIO) mice and normal mice. The results showed that the expression of miR-126b-5p in obese mice was approximately four times that of normal mice (Figure 1B). At the same time, the expression of miR-126b-5p was determined in the inguinal adipose tissue of 1–6-week-old mice. As shown in Figure 1C, the expression of miR-126b-5p in 1–6 weeks-old mice inguinal adipose tissues increased gradually. In addition to determining the expression of miR-126b-5p in vivo, we also evaluated it in the process of 3T3-L1 preadipocyte differentiation. During the process of 3T3-L1 preadipocyte differentiation, the expression of miR-126b-5p increased gradually (Figure 1D). These findings proved that miR-126b-5p is associated with adipogenesis, possibly playing an essential role in the process.

### 2.2. miR-126b-5p Promotes 3T3-L1 Preadipocyte Proliferation and Differentiation

**Figure 2:**
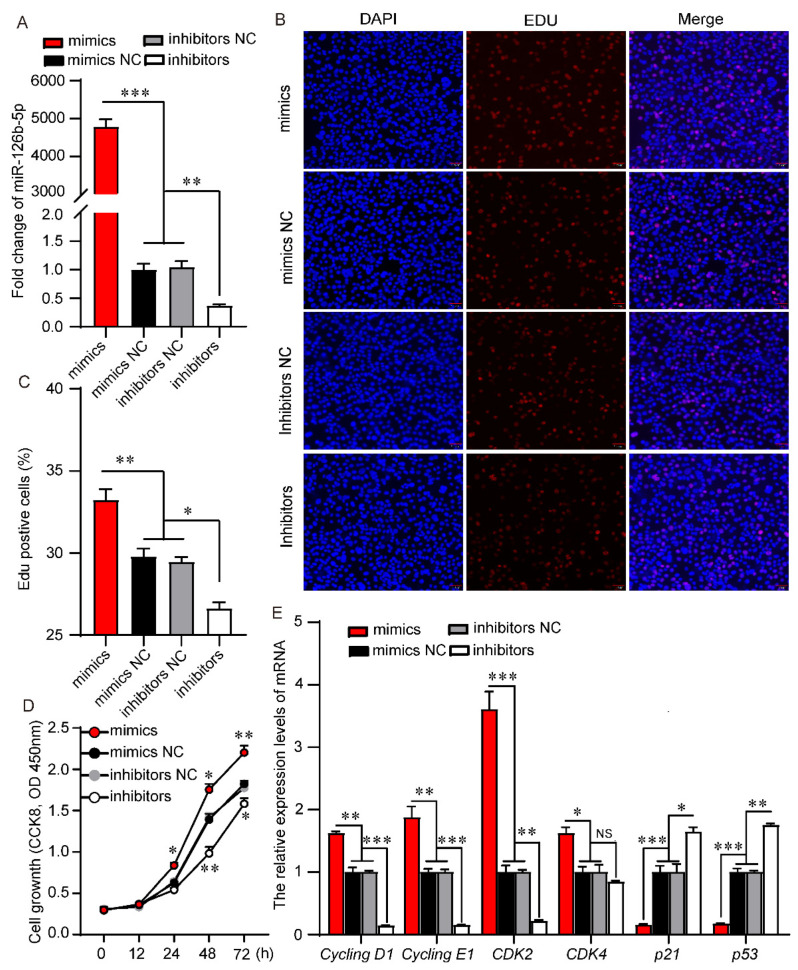
miR-126b-5p promoted proliferation of 3T3-L1 preadipocytes. (**A**) The expression of miR-126b-5p after transfecting miR-126b-5p mimics, NC mimics, NC inhibitors, and miR-126b-5p inhibitors at 48 h. The EdU assay (**B**), quantification of EdU positive cells (**C**), and CCK8 assay (**D**) were used to evaluate the proliferation of 3T3-L1 preadipocytes. (**E**) The expression of *Cycling D1*, *Cycling E1*, *CDK2*, *CDK4*, *p21*, and *p53* 48 h after transfection of miR-126b-5p mimics, NC mimics, NC inhibitors, and miR-126b-5p inhibitors (scale bars, 200 μm). The data are presented as mean ± SEM (*n* = 3) (NS—not significant, *p* > 0.05, * *p* < 0.05, ** *p* < 0.01, *** *p* < 0.001).

To investigate the role of miR-126b-5p in 3T3-L1 preadipocyte proliferation, the miR-126b-5p mimics, miR-126b-5p inhibitors, NC mimics, and NC inhibitors were transfected into 3T3-L1 preadipocytes during the proliferation process. As shown in Figure 2A, the expression level of miR-126b-5p after the transfection of miR-126b-5p mimics was significantly higher than NC mimics, and the expression level of transfection miR-126b-5p inhibitors was significantly lower than NC inhibitors, indicating that the transfection was successful. The CCK8 and EdU assays are common methods to assess cell proliferation. Subsequently, we used CCK8 and EdU assays to evaluate the effect of miR-126b-5p on the proliferation of 3T3-L1 preadipocytes. The EdU results showed that transfecting miR-126b-5p mimics increased EdU-positive cells and transfecting miR-126b-5p inhibitors decreased EdU-positive cells (Figure 2B,C). Similar to EdU assay results, the CCK8 assay showed no change in the cell growth rate after transfecting miR-126b-5p mimics or inhibitors at 12 h, but it significantly increased and decreased at the late proliferation stage, respectively (Figure 2D). The expression of proliferation-related genes is also compelling evidence to evaluate cell proliferation. Therefore, we determined the expression level of cell cycle factors (*Cycling D1*, *Cycling E1*, *CDK2*, and *CDK4*) and cell apoptotic factors (*p21* and *p53*). The results showed that the transfection of miR-126b-5p mimics or miR-126b-5p inhibitors could significantly increase or decrease the expression of *Cycling D1*, *Cycling E1*, *CDK2*, and *CDK4*, respectively, and the effects on *p21* and *p53* were the opposite (Figure 2E).

**Figure 3:**
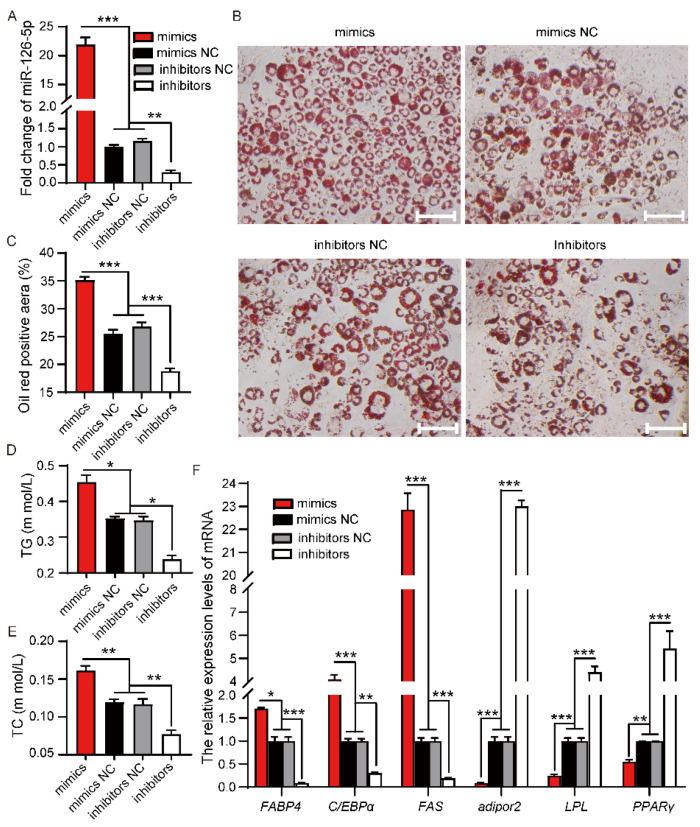
miR-126b-5p enhanced the lipid deposition of 3T3-L1 preadipocytes. (**A**) The expression of miR-126b-5p after transfecting miR-126b-5p mimics, NC mimics, NC inhibitors, and miR-126b-5p inhibitors eight days after 3T3-L1 differentiation. (**B**–**E**) The Oil Red O staining (**B**), quantification of Oil Red O staining-positive area (**C**), the content of TAG (**D**), and TC (**E**) detection were used to evaluate the differentiation of 3T3-L1 preadipocytes. (**F**) The expression of *FABP4*, *C/EBPα*, FAS, *adipor2*, *LPL*, and *PPARγ* after transfecting miR-126b-5p mimics, NC mimics, NC inhibitors, and miR-126b-5p inhibitors eight days after 3T3-L1 differentiation (scale bars, 200 μm). The data are presented as mean ± SEM (*n* = 3) (* *p* < 0.05, ** *p* < 0.01, *** *p* < 0.001).

Considering the expression pattern of miR-126b-5p during fat development in vivo and vitro, it was speculated that miR-126b-5p could promote 3T3-L1 differentiation. Analogously, we transfected the miR-126b-5p mimics, miR-126b-5p inhibitors, NC mimics, and NC inhibitors into 3T3-L1 preadipocytes in the differentiation process. As shown in Figure 3A, the expression of miR-126b-5p after transfecting miR-126b-5p mimics increased significantly compared with NC mimics, and transfection of miR-126b-5p inhibitors significantly decreased compared with NC inhibitors. Subsequently, Oil Red O staining was used to clarify the effect of miR-126b-5p on the differentiation of 3T3-L1 preadipocytes. As shown in Figure 3B, transfecting miR-126b-5p mimics promoted the accumulation of lipid droplets noticeably, and transfecting miR-126b-5p inhibitors inhibited lipid droplet accumulation. The quantification of the Oil Red O staining-positive area indicated that miR-126b-5p mimics significantly promoted lipid droplet accumulation (Figure 3C). Moreover, the TG and TC levels were investigated after transfection of miR-126b-5p mimics. As shown in Figure 3D,E, miR-126b-5p mimics could increase the TG and TC content in 3T3-L1 preadipocytes, and miR-126b-5p inhibitors exhibited an opposite effect. Finally, the expression of genes related to lipid metabolism was detected. Consistently, miR-126b-5p mimics promoted the expression of adipogenic genes and inhibited the expression of lipid catabolism genes.

As shown in Figure 3F, the expression of many fatty-acid-metabolism-related genes had changed. Therefore, it was suspected that the composition and content of fatty acids in 3T3-L1 preadipocytes had also changed. In addition to verifying this conjecture, the composition and content of fatty acids in 3T3-L1 preadipocytes were analyzed after inducing differentiation for eight days, using gas chromatography–mass spectrometry. As shown in Table 1, the capric acid (C10:0) and lauric acid (C12:0) emerged after transfecting miR-126b-5p mimics, and the concentrations of tridecanoic acid (C13:0), myristic acid (C14:0), pentadecanoic acid (C15:0), palmitic acid (C16:0), palmitoleic acid (C16:1), heptadecanoic acid (C17:0), 10-cis-heptadecanoic acid (C17:1), oleic acid (C18:1n9c), stearic acid (C18:0), and gamma-linolenic acid (C18:3n6) increased significantly, compared with NC mimics. However, the concentrations of linoleic acid (C18:2n6c), eicosanoic acid (C20:0), eicosatrienoic acid (C20:3n6), arachidonic acid (C20:4n6), eicosapentaenoic acid (C20:5n3), behenic acid (C22:0), docosahexaenoic acid (C22:6), and lignoceric acid (C24:0) exhibited a nonsignificant upward trend. Among all the fatty acids, only the eicosadienoic acid (C20:2) concentration decreased, which was not significant. Subsequently, the concentrations of total fatty acids, saturated fatty acid (SFA), monounsaturated fatty acid (MUFA), and polyunsaturated fatty acid (PUFA) were analyzed. The results showed that the total fatty acid content was significantly upregulated, which might have been caused by the remarkable upregulation of the SFA and MUFA content. Moreover, the C16:0/C18:0, C16:1/C18:1, SFA/total, and MUFA/total ratios were significantly upregulated in the H-126 group, and C18:0/C18:1 and PUFA/total ratios were significantly downregulated. The ratio of C16:0/C16:1 had no significant change. 

### 2.3. Adipor2 and ACADL Are Direct Target Genes of miR-126b-5p

**Figure 4:**
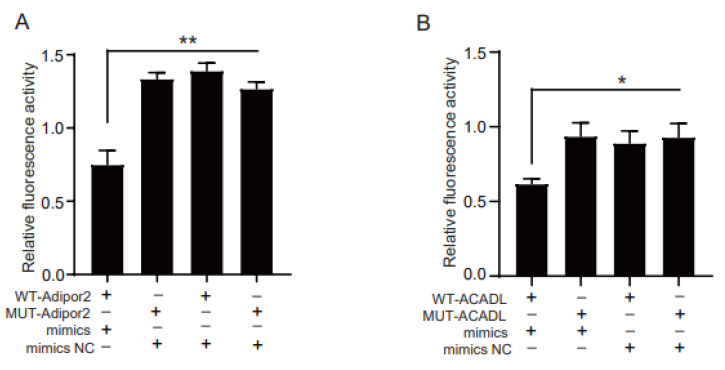
*Adipor2* and *ACADL* are direct target genes of miR-126b-5p measured in HEK-293T cells. (**A**,**B**) The repressive effect of miR-126b-5p on the activity of Adipor2 and ACADL 3′UTR was measured by the luciferase assay. The data are presented as mean ± SEM (*n* = 3) (* *p* < 0.05, ** *p* < 0.01, *** *p* < 0.001).

At present, most studies have shown that miRNAs silence or degrade target genes by binding their 3′UTR region. To clarify the potential mechanism of miR-126b-5p regulating 3T3-L1 preadipocyte differentiation, TargetScan (http://www.targetscan.org/, 1 June 2021) and miRDB (http://www.mirdb.org/, 1 June 2019) were used to predict the target genes of miR-126b-5p. In the present study, we focused on determining whether the target genes of miR-126b-5p were related to adipogenesis. Fortunately, we successfully screened Adipor2 and ACADL from the target genes list, which might involve adipogenesis. Next, the luciferase reporter experiment was used to verify whether miR-126b-5p had a direct target relationship with the two genes. Consistent with this expectation, the group of co-transfecting miR-126b-5p mimics and wild-type 3′UTR of the target gene significantly inhibited the fluorescence activity (Figure 4A,B).

### 2.4. miR-126b-5p Exacerbated Obesity Induced by a High-Fat Diet

**Figure 5:**
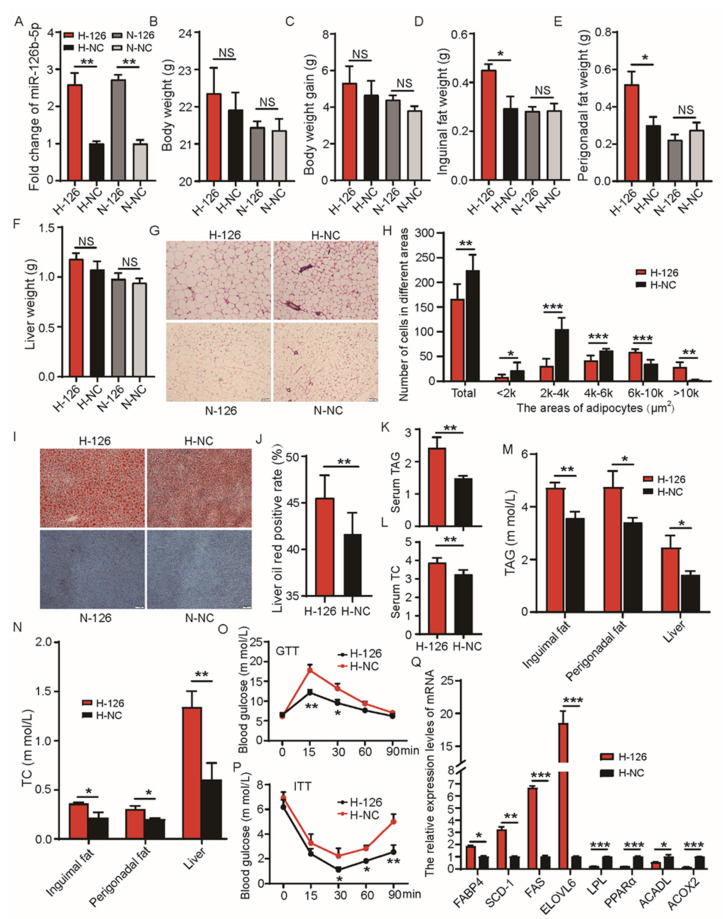
miR-126b-5p exacerbated mice obesity when induced by a high-fat diet. (**A**) The expression of miR-126b-5p in the inguinal adipose tissue was persistently upregulated for eight weeks. The body weight (**B**), body weight gain (**C**), inguinal fat weight (**D**), perigonadal fat weight (**E**), and liver weight (**F**) in the four groups. (**G**) The H&E staining of the inguinal adipose tissue in the four groups. (**H**) The quantitation for the cell size and number of perigonadal fat in the H-126 and H-NC groups. The Oil Red O staining of the liver (**I**) and the statistics of Oil Red O staining positivity rate (**J**) in the liver. The concentration of TAG (**K**) and TC (**L**) in the serum. The concentration of TAG (**M**) and TC (**N**) in inguinal adipose tissue, perigonadal adipose tissue, and liver. The glucose tolerance test, GTT (**O**) and insulin tolerance test, ITT (**P**). (**Q**) The expression of *FABP4*, *SCD-1*, *FAS*, *ELOVL6*, *LPL*, *PPARα*, *ACADL*, and *ACOX2* in the inguinal adipose tissue. All the results were presented as mean ± SEM (*n* = 4–6, Scale bars, 100 μm) (NS—not significant, *p* > 0.05, * *p* < 0.05, ** *p* < 0.01, *** *p* < 0.001).

To verify whether miR-126b-5p influenced lipid deposition in vivo, we persistently overexpressed miR-126b-5p in C57BL/6 mice for eight weeks. Firstly, the expression of miR-126b-5p was detected in the inguinal adipose tissue of four groups of mice. As shown in Figure 5A, the expressions of miR-126b-5p in the H-126 and N-126 groups was noticeably higher than in the H-NC and N-NC groups. These findings indicated that overexpression models were successfully constructed. Subsequently, the body weight, body weight gain, and liver weight were observed in the H-126 and H-NC groups. This indicated no significant change, but the H-126 group exhibited an upward trend compared to the H-NC group. Moreover, the inguinal fat weight and perigonadal fat weight in the H-126 group were significantly higher than in the H-NC group (Figure 5B–F). However, these changes did not occur in the N-126 and N-NC groups (Figure 5B–F). Then, the H&E and Oil Red O staining were performed to evaluate the changes in the size and number of inguinal adipose tissue cells and the liver lipid accumulation, respectively. As shown in Figure 5G,H, the cells in the H-126 group were notably bigger than the H-NC group. However, the cell sizes and the number of cells in the N-126 group showed no change compared to the N-NC group, consistent with the unchanged inguinal fat weight between the N-126 and N-NC groups (Figure 5G). As shown in Figure 5I,J, the liver Oil Red O staining-positive area of the H-126 group was significantly higher than that in the H-NC group. Similarly, the liver tissues of the N-126 and N-NC groups were not positive for Oil Red O staining, indicating that overexpression of miR-126b-5p might play a role in a high-fat diet but not in a normal chow diet.

Subsequently, all the analyses were performed in the high-fat diet group. The content of TAG and TC is an important lipid deposition indicator. Therefore, the effect of miR-126b-5p overexpression on TAG and TC content was evaluated in different tissues and serum. As shown in Figure 5K–N, the concentrations of TAG and TC in serum, inguinal adipose tissue, perigonadal adipose tissue, and liver in the H-126 group were notably higher than those in the H-NC group. It is well established that insulin resistance is one of the crucial characteristics in evaluating obesity. Therefore, the glucose tolerance test (GTT) and insulin tolerance test (ITT) were performed in this study to confirm the effect of miR-126b-5p on insulin resistance. The results showed that the H-126 group had a higher blood glucose level than the H-NC group in GTT and ITT (Figure 5O,P), indicating that miR-126b-5p overexpression impaired glucose homeostasis and insulin sensitivity. Finally, the expression of lipid-deposition-related genes was determined in the inguinal adipose tissue. The same preadipocyte experiment showed that the expression of *FABP4*, *SCD-1*, *FAS*, and *ELOVL6* significantly increased, but the expression of *LPL*, *PPARα*, *ACADL*, and *ACOX2* significantly decreased when the H-126 group was compared to the H-NC group (Figure 5Q). These findings demonstrated that overexpression of miR-126b-5p in the mice fed with a high-fat diet exacerbated obesity.

In addition, the composition and content of fatty acids in the inguinal adipose tissue were determined in the H-126 and H-NC groups. As shown in Table 2, the contents of total fatty acids, SFA, MUFA, and PUFA in the H-126 group showed no change compared to the H-NC group. The contents of C8:0, C10:0, C14:0, C15:1, C16:1, C18:0, C20:0, C20:4n6, C22:0, C22:2n6, C23:0, and C24:0 were notably upregulated in the H-126 group compared with the H-NC group. However, the contents of C6:0, C11:0, C12:0, C13:0, C14:1, C15:0, C16:0, C17:0, C18:1n9c, C18:2n6c, C20:1, C20:3n6, C20:3n3, C22:2n9, C24:1, and C22:6 showed no change between H-126 and H-NC groups. The contents of C20:2 and C20:5n3 were significantly downregulated in the H-NC group. Subsequently, we also analyzed the ratio changes of C16:0/C16:1, C16:0/C18:0, C18:0/C18:1, C16:1/C18:1, SFA/total, MUFA/total, and PUFA/total. As shown in Table 2, the C16:0/C16:1 and C16:0/C18:0 ratios were significantly downregulated in the H-126 group, and the C18:0/C18:1 and C16:1/C18:1 ratios were significantly upregulated. The SFA/total, MUFA/total, and PUFA/total ratios showed no changes.

## 3. Discussion

Obesity has become an epidemic, seriously endangering human health. The reason for obesity is that energy intake is greater than metabolic consumption. White adipose tissue (WAT) is the main location for storing energy and converts carbohydrates in food into triglycerides [15]. However, the ability of white adipose tissue to store energy is limited. Excessive triglyceride accumulation in white adipose tissue causes obesity, leading to a series of diseases such as T2DM, non-alcoholic fatty liver, and cardiovascular diseases [1,2,3,4]. In recent years, more and more miRNAs have been reported to be associated with obesity. However, miR-126b-5p has not been reported in the field of obesity. The present study demonstrated that the *mmu-miR-126b-5p* is associated with adipogenesis. Therefore, it is necessary to study the role of miR-126b-5p in the obesity process.

The quality of adipose tissue is mainly determined by the number and size of adipocytes [16]. The number of adipocytes is regulated by proliferation-related genes, and the size of adipocytes is determined by their lipid content. The 3T3-L1 preadipocytes were used to assess the effect of miR-126b-5p on adipocyte proliferation and differentiation in vitro, and the results showed that miR-126b-5p could promote the proliferation and differentiation of 3T3-L1 preadipocytes. In previous studies, the role of cell-cycle-related genes has been investigated in depth. For example, the cell cycle was arrested in the G1-S transition period in the absence of *CDK2* [17]. *p21* is an inhibitor of *CDK*/cyclin complexes and has the most obvious inhibitory effect on *CDK2* [18,19]. *p53* can arrest the cell cycle by regulating the expression of *p21* [20]. In the present study, the overexpression of miR-126b-5p inhibited the expression of *p21* and *p53* and promoted the expression of *cycling D1*, *cycling E1*, *CDK2*, and *CDK4*. Inhibiting the expression of miR-126b-5p resulted in opposite results.

Moreover, too many genes have been reported to be involved in differentiation, and *PPAR* and *C/EBP* families play an important role in it [21,22]. In a previous study, *Adipor2* was inversely proportional to the degree of obesity and was identified as a potential therapeutic target for treating obesity [23]. Activating hepatic *Adipor2* could protect mice against non-alcoholic steatohepatitis, and *Adipor2* deficiency could promote T2DM [24,25]. Kraus et al. reported that miR-375 knockdown could inhibit adipogenic differentiation by directly targeting *Adipor2* [26]. The *ACADL* gene encodes long-chain acyl-CoA dehydrogenase (*LCAD*), which plays a role in β-oxidation of fatty acids with 12–16 carbon atoms in mitochondria [27]. A lack of *ACADL* resulted in the accumulation of diacylglycerol, liver insulin resistance, and myocardial hypertrophy [28,29,30]. All the above studies have demonstrated that *Adipor2* and *ACADL* are oppositely regulated in adipogenesis and lipogenesis. In the present study, *Adipor2* and *ACADL* were identified as the direct target genes of miR-126b-5p, proving that miR-126b-5p has a promoting effect on adipogenesis. Interestingly, most previous studies have shown that miRNA generally inhibits proliferation and promotes differentiation [7,8,9]; however, miR-126b-5p promoted both proliferation and differentiation in the present study. Cell proliferation and differentiation is a complex and orderly process. Perhaps, miR-126b-5p promotes proliferation, leading to an increased number of cells and increased lipid accumulation in them, further exacerbating obesity.

Fatty acids are important intermediate products of lipid metabolism. Fatty acids are usually combined in groups of three to form triglycerides for energy storage; they can also provide energy for the body through the β-oxidation pathway [31]. The present study showed that the overexpression of miR-126b-5p significantly increased the total fatty acid content in vitro. The SFA/total and MUFA/total ratios were significantly higher, and the PUFA/total ratio was significantly lower in the miR-126b-5p mimics group compared with the NC mimics group. Moreover, the C16:0/C16:1 and C16:0/C18:0 ratios in vivo were significantly downregulated, and the C16:1/C18:1 and C18:0/C18:1 ratios were significantly upregulated. These findings were consistent with the high expression of SCD-1 and ELOVL6. The SCD-1 gene mainly regulates the conversion of saturated fatty acids to unsaturated fatty acids, and the conversions of C16:0 and C18:0 to C16:1 and C18:1 are the two main processes of SCD-1 gene regulation [32]. The ELOVL6 gene mainly catalyzes C16:0 elongation of the carbon chain to generate C18:0 [33], consistent with the present study results. However, different results were obtained at the cellular level. The C16:0/C16:1 and C18:0/C18:1 ratios were downregulated, and the C16:1/C18:1 and C16:0/C18:0 ratios were upregulated in 3T3-L1 preadipocytes because the expression of *SCD-1* increased significantly after transfecting miR-126b-5p; however, the expression of *ELOVL6* did not increase (data not shown). On the other hand, the expression of FAS which promotes the de novo synthesis of C16:0 increased significantly. Therefore, although the cellular level can reflect part of the in vivo results, there are still differences.

Finally, we observed that miR-126b-5p overexpression in vivo aggravated obesity. In the present study, the inguinal and perigonadal fat weights significantly increased in the H-126 group compared with the H-NC group. The Oil Red O-positive rate in the liver tissue of the H-126 group and the cell size of inguinal adipose tissue increased significantly. Moreover, the TAG and TC levels in the serum and some tissues of the H-126 group mice also increased significantly, indicating that the metabolism was aggravated, and hyperlipidemia was more serious. Glucose tolerance and insulin resistance are also important indicators for evaluating obesity [34]. In the present study, the GTT and ITT assay showed that the H-126 group mice had worse glucose tolerance and insulin sensitivity, indicating that the overexpression of miR-126b-5p aggravated obesity in C57BL/6 mice; this also suggests that inhibiting the expression of miR-126b-5p is a significant potential target for inhibiting obesity.

## 4. Materials and Methods

### 4.1. Animal Treatment and Sample Collection

All the experimental mice were purchased from Chengdu Dossy Experimental Animals Co., Ltd., China. The animal experiments were approved by the Institutional Animal Care and Use Committee of the College of Animal Science and Technology of Sichuan Agricultural University, Sichuan, China (approval number: 20200038).

The inguinal adipose tissue of high-fat diet (HFD)-treated mice and regular chow diet (NCW) mice were obtained from our previous study. Eleven different tissue profiles were obtained from three 8-week-old female C57BL/6J mice. Samples were obtained at different developmental stages (1–6 weeks old) from 18 female C57BL/6J mice. All the samples were collected quickly and stored at −80 °C. Another 24 mice (6 weeks old, after temporary feeding for one week to adapt to the environment) were randomly divided into four groups. Randomly, two groups were fed an HFD (feed ingredients: water, 93 g/kg; protein, 134 g/kg; fat, 143 g/kg; total sugar, 235 g/kg; crude fiber, 27 g/kg; calcium, 8.3 g/kg; total phosphorus, 7.1 g/kg; and the rest consisted of starch), while the other two groups were fed an NC (feed ingredients: water, 94 g/kg; protein, 190 g/kg; fat, 51 g/kg; crude fiber, 36 g/kg; ash, 62 g/kg; calcium, 11.3 g/kg; total phosphorus, 8.6 g/kg; and the rest consisted of starch). One HFD group and one NC group were randomly selected to overexpress miR-126b-5p by injecting pre-miR-126b-5p plasmid intraperitoneally, as H-126 and N-126 groups, and the other groups were injected the same amount of normal saline solution, as H-NC and N-NC groups, respectively, to explore the effect of miR-126b-5p on adipogenesis in vivo. The in vivo overexpression experiments by intraperitoneal injection of plasmids were based on previous studies [35,36,37]. The concentration of pre-miR-126b-5p plasmid injection was controlled at 1000 μg/μL for every mouse. The mice were euthanized after two months of treatment. The mice were free to eat and drink and were fed under natural light and dark cycles with the ambient temperature controlled at 22–25 °C. The collection of adipose tissues and liver samples was consistent with the above description.

### 4.2. Cell Culture and Transfection

The HEK293T cells and 3T3-L1 preadipocytes (from the China Infrastructure of Cell Line Resource, Beijing, China) were cultured in an incubator containing 5% CO_2_ and kept at 37 °C in the growth medium (GM). The GM contained Dulbecco’s modified Eagle’s medium (DMEM, Gibco, Carlsbad, CA, USA) and 10% fetal bovine serum (FBS, Gibco). The classic “cocktail” method was used to differentiate 3T3-L1 preadipocytes. The 3T3-L1 cells were cultured to 70–80% density and spread on the plates. When the corresponding density was reached, the medium was converted to differentiation medium (DM)-A for three days. Subsequently, DM-B and GM were cultured for three days, respectively. The DM-A contained DMEM, 10% FBS, 0.5-nM 3-isobutyl-1-ethylxanthine (IBMX), 1-nM dexamethasone, and 5 mg/mL of insulin; DM-B consisted of DMEM, 10% FBS, and 5 mg/mL insulin.

For transfection, cells were transfected with miR126b-5p mimics, miR-126b-5p inhibitor, NC mimics, and NC inhibitor (Ribobio, Guangzhou, China) using lipofectamine 3000 (Lipo 3000, Invitrogen, Carlsbad, CA, USA), according to the manufacturer’s instructions. Taking the 12-well plates with 1 mL of the medium as an example, first, 100 μL of opti-MEM was incubated with 5 μL of transfection reagent and Lipo 3000, separately, for 5 min. Subsequently, they were mixed and incubated for 10 min and added to the culture medium to transfect cells.

### 4.3. Proliferation Analysis

Cell Counting Kit 8 (CCK-8, Beyotime, Shanghai, China) and 5-ethynyl-20-deoxyuridine (EdU, Ribobio, Guangzhou, China) assays were used in the present study to detect the proliferation of 3T3-L1 cells. After 3T3-L1 preadipocytes were seeded in 96-well plates and transfected with the target reagent, 10 μL of CCK-8 was added to each well. The absorbance was detected at 450 nm wavelength, 0, 12, 24, 48, and 72 h after incubating for 1 h. For the EdU assay, the pre-configured EdU reagent with a concentration of 50 μM was added to 96-well plates, and after incubating for 2 h, the cells were fixed with 4% paraformaldehyde for 30 min, washed with phosphate-buffered saline solution (PBS, Gibco), and stained with EdU kit. Finally, the images were obtained with a Nikon TE2000 microscope (Nikon, Tokyo, Japan).

### 4.4. Triglyceride, Total Cholesterol Assay, and Cell Oil Red O Staining

The triglyceride (TG) and total cholesterol (TC) assays were performed according to the manufacturer’s instructions (Nanjing Jiancheng Bioengineering Institute, Nanjing, China). The cell sample underwent ultrasonic vibration to disintegrate the cell under ice-water-bath condition after adding 0.2–0.3 mL of PBS; then, the homogenized lipid could be directly measured. The 30 mg tissue samples were mechanically homogenized under ice-water-bath conditions after adding nine-times volume of absolute ethanol. Then the samples were centrifuged at 2500 rpm/min for 10 min, and the supernatant was collected to be measured. The serum samples could be directly measured. For Oil Red O staining, the 3T3 cells differentiated for eight days were fixed with 4% paraformaldehyde for 30 min. Subsequently, the cells were soaked in 60% isopropanol solution for 15 s and washed with PBS. Finally, the cells were stained with Oil Red O staining solution (Oil Red O stock solution: isopropanol = 3:2).

### 4.5. Tissue H&E and Oil Red O Staining

First, the inguinal adipose tissue was fixed with 4% paraformaldehyde, and then the sample was dehydrated, embedded, sliced, and stained with hematoxylin and eosin (H&E) to obtain images. Concerning Oil Red O staining, the frozen sections of mouse liver tissue were prepared and stained with Oil Red O. Finally, images were obtained after preparing the sections.

### 4.6. Glucose Tolerance Test (GTT) and Insulin Tolerance Test (ITT)

For the glucose tolerance test (GTT), 1.5 g/kg (glucose/body weight) of glucose solution was injected intraperitoneally after 12 h of fasting. For the insulin tolerance test (ITT), 0.75 U/kg (insulin/body weight) of insulin solution was also injected intraperitoneally after 4 h of fasting. Blood samples were collected from the mouse’s tail vein after injection at 0, 15, 30, 60, and 90 min and detected by a blood glucose meter (Accu-Check, Roche, Shanghai, China).

### 4.7. Analysis of Fatty Acids

The composition and content of fatty acids were detected by gas chromatography-mass spectrometry; 50 mg of inguinal adipose tissues or cell samples were collected, and the test was completed by Beijing Masspeaks Technology Co., Ltd., Beijing, China.

### 4.8. Quantitative Real-Time PCR

Total RNA was extracted from the cells and tissues using TRIzol Reagent (TaKaRa, Dalian, China) according to the manufacturer’s instructions. Subsequently, cDNAs were generated by reverse transcription, and quantitative real-time PCR was performed by the SYBR Premix Ex Taq kit (TaKaRa; Dalian, China) on a CFX96 system (Bio-Rad, Hercules, CA, USA). Relative expression levels of mRNAs and miRNAs were calculated using the 2^−ΔΔCt^ method. In addition, the expression levels of *U6* and *β-actin* were used as endogenous controls to normalize the expression of miRNA and mRNA, respectively. The primer sequences and annealing temperature used for qRT-PCR are listed in Table 3.

### 4.9. Luciferase Reporter Assay

The wild-type 3′ UTR of *Adipor2* and *ACADL* (WT-*Adipor2* and WT-*ACADL*) and mutant-type 3′ UTR of *Adipor2* and *ACADL* (MUT-*Adipor2* and MUT-*ACADL*), constructed by Tsingke Biotechnology Co., Ltd. (Chengdu, China), were cloned into psiCHECK^TM^-2 vectors. For luciferase reporter assay, the WT or MUT 3′ UTR was co-transfected with miR-126b-5p mimics or NC mimics into HEK-293T cells using lipofectamine 3000. The cells were collected 48 h after transfection, and luciferase activities were measured by Dual-Glo Luciferase Assay System (Promega, Madison, WI, USA) following the manufacturer’s instructions.

### 4.10. Statistical Analysis

All the experimental data were reported as mean ± SEM (standard error of the mean) and analyzed using SPSS 27.0. The significance of the difference was analyzed using Student’s *t*-test. *p* < 0.05 indicated that the data were statistically significant (N.S. *p* > 0.05, * *p* < 0.05, ** *p* < 0.01, *** *p* < 0.001).

## 5. Conclusions

Taken together, the present study showed that miR-126b-5p promoted the proliferation and differentiation of 3T3-L1 preadipocytes. miR-126b-5p also increased the fatty acid content and promoted lipid deposition in vivo and in vitro. The results of the work suggest that miR-126b-5p participates in the regulation of genes involved in adipocyte proliferation and differentiation, exacerbating obesity. Therefore, this miRNA emerges as a new therapeutic target to delay or decelerate adipogenesis in obesity.

## Figures and Tables

**Table 1:** miR-126b-5p affected the composition of fatty acids in 3T3-L1 preadipocytes.

Fatty Acid	Concentration (μg/mL)		Up/Down
	Mimics NC	miR-126b-5p Mimics	
C6:0	0	0	----
C8:0	0	0	----
C10:0	0	0.01 ± 0.0004	Up
C11:0	0	0	----
C12:0	0	0.02 ± 0.002	Up
C13:0	0.01 ± 0.00	0.02 ± 0.001	Up ***
C14:0	0.54 ± 0.05	1.23 ± 0.04	Up ***
C14:1	0	0	----
C15:0	0.46 ± 0.05	1.01 ± 0.02	Up ***
C15:1	0	0	----
C16:0	8.29 ± 0.38	18.16 ± 0.13	Up ***
C16:1	4.57 ± 0.44	11.01 ± 0.55	Up ***
C17:0	0.42 ± 0.04	0.77 ± 0.02	Up ***
C17:1	0.54 ± 0.05	1.10 ± 0.09	Up ***
C18:0	3.60 ± 0.76	5.02 ± 0.03	Up *
C18:1n9t	0	0	----
C18:1n9c	5.20 ± 0.71	10.02 ± 0.56	Up ***
C18:2n6t	0	0	----
C18:2n6c	0.52 ± 0.04	0.55 ± 0.02	Up ^NS^
C18:3n6	0.25 ± 0.001	0.26 ± 0.01	Up *
C18:3n3	0	0	----
C20:0	0.20 ± 0.01	0.21 ± 0.01	Up ^NS^
C20:1	0	0	----
C20:2	0.68 ± 0.0005	0.66 ± 0.05	Down ^NS^
C21:0	0	0	----
C20:3n6	0.484 ± 0.03	0.485 ± 0.02	Up ^NS^
C20:4n6	1.61 ± 0.23	1.65 ± 0.11	Up ^NS^
C20:3n3	0	0	----
C20:5n3	0.53 ± 0.05	0.55 ± 0.04	Up ^NS^
C22:0	0.168 ± 0.005	0.174 ± 0.003	Up ^NS^
C22:1n9	0	0	----
C22:2n6	0	0	----
C23:0	0	0	----
C24:0	0.25 ± 0.01	0.26 ± 0.005	Up ^NS^
C24:1	0	0	----
C22:6	0.846 ± 0.05	0.853 ± 0.03	Up ^NS^
total	29.17 ± 2.60	54.02 ± 0.97	Up ***
SFA	13.94 ± 1.09	26.88 ± 0.02	Up ***
MUFA	10.3 ± 1.15	22.13 ± 1.20	Up ***
PUFA	4.92 ± 0.40	5.01 ± 0.25	Up ^NS^
C16:0/C16:1	1.82 ± 0.090	1.65 ± 0.094	Down ^NS^
C16:0/C18:0	2.37 ± 0.49	3.62 ± 0.044	Up *
C18:0/C18:1	0.69 ± 0.057	0.50 ± 0.026	Down **
C16:1/C18:1	0.89 ± 0.074	1.10 ± 0.0070	Up **
SFA/total	0.48 ± 0.0065	0.50 ± 0.0091	Up *
MUFA/total	0.35 ± 0.0081	0.41 ± 0.015	Up **
PUFA/total	0.17 ± 0.0068	0.093 ± 0.0061	Down ***

Saturated fatty acid (SFA) = C6:0 + C8:0 + C10:0 + C11:0 + C12:0 + C13:0 + C14:0 + C15:0 + C16:0 + C17:0 + C18:0 + C20:0 + C21:0 + C22:0 + C23:0 + C24:0. Monounsaturated fatty acid (MUFA) = C14:1 + C15:1 + C16:1 + C17:1 + C18:1n9t + C18:1n9c + C20:1 + C22:1n9 + C24:1. Polyunsaturated fatty acid (PUFA) = C18:2n6t + C18:2n6c + C18:3n3 + C18:3n6 + C20:2 + C20:3n6 + C20:4n6 + C20:3n3 + C22:5n3 + C22:2n6 + C22:6. Data are presented as mean ± SEM (*n* = 3) (NS—not significant, *p* > 0.05, * *p* < 0.05, ** *p* < 0.01, *** *p* < 0.001).

**Table 2:** miR-126b-5p regulated the composition of fatty acids in inguinal adipose tissue.

Fatty Acid	Concentration (μg/g)		Up/Down
	H-NC	H-126	
C6:0	27.84 ± 2.75	28.85 ± 0.66	NS
C8:0	9.84 ± 0.19	11.82 ± 0.80	Up *
C10:0	49.24 ± 2.76	68.67 ± 3.51	Up **
C11:0	1.04 ± 0.14	1.19 ± 0.02	NS
C12:0	299.48 ± 25.58	335.03 ± 35.38	NS
C13:0	6.11 ± 0.74	6.86 ± 0.02	NS
C14:0	6586.37 ± 365.78	7352.71 ± 133.51	Up *
C14:1	320.40 ± 29.42	342.04 ± 5.08	NS
C15:0	341.88 ± 11.10	333.69 ± 5.41	NS
C15:1	0.75 ± 0.27	2.01 ± 0.13	Up **
C16:0	67,993.57 ± 1538.06	64,103.13 ± 3242.12	NS
C16:1	21,977.17 ± 844.14	25,118.60 ± 333.46	Up **
C17:0	622.01 ± 26.90	636.70 ± 2.65	NS
C17:1	836.88 ± 6.36	835.63 ± 17.52	NS
C18:0	20,838.49 ± 9.99	26,268.43 ± 227.55	Up ***
C18:1n9t	0	0	----
C18:1n9c	118,554.11 ± 1652.67	117,502.15 ± 3730.93	NS
C18:2n6t	0	0	----
C18:2n6c	60,435.67 ± 402.57	58,232.17 ± 2572.42	NS
C18:3n6	0	0	----
C18:3n3	4034.85 ± 94.16	4145.20 ± 39.24	NS
C20:0	576.07 ± 29.08	646.11 ± 6.99	Up *
C20:1	3601.34 ± 35.07	3583.60 ± 19.50	NS
C20:2	1420.30 ± 12.40	1459.13 ± 4.35	Down **
C21:0	0	0	----
C20:3n6	536.93 ± 22.31	556.34 ± 1.11	NS
C20:4n6	685.24 ± 10.66	821.83 ± 46.59	Up **
C20:3n3	200.46 ± 5.48	201.28 ± 1.71	NS
C20:5n3	182.35 ± 13.88	154.11 ± 1.73	Down *
C22:0	95.49 ± 3.51	103.27 ± 1.26	Up *
C22:1n9	170.41 ± 0.57	171.81 ± 3.16	Up ^NS^
C22:2n6	8.83 ± 0.33	10.20 ± 0.19	Up **
C23:0	15.42 ± 1.45	19.39 ± 1.35	Up *
C24:0	34.29 ± 8.86	64.93 ± 4.91	Up **
C24:1	107.11 ± 4.00	106.56 ± 0.61	NS
C22:6	905.71 ± 80.71	876.80 ± 49.26	NS
total	311,475.65 ± 4127.86	314,100.24 ± 2094.52	NS
SFA	97,497.15 ± 1739.71	99,980.78 ± 3565.31	NS
MUFA	145,568.17 ± 2210.44	147,662.40 ± 4110.32	NS
PUFA	68,410.34 ± 519.11	66,457.06 ± 2638.75	NS
C16:0/C16:1	3.10 ± 0.10	2.55 ± 0.16	Down **
C16:0/C18:0	3.26 ± 0.073	2.44 ± 0.10	Down ***
C18:0/C18:1	0.18 ± 0.0024	0.22 ± 0.0090	Up ***
C16:1/C18:1	0.19 ± 0.0063	0.21 ± 0.0040	Up **
SFA/total	0.31 ± 0.0016	0.32 ± 0.0092	NS
MUFA/total	0.47 ± 0.0023	0.47 ± 0.016	NS
PUFA/total	0.22 ± 0.0024	0.21 ± 0.0070	NS

SFA = C6:0 + C8:0 + C10:0 + C11:0 + C12:0 + C13:0 + C14:0 + C15:0 + C16:0 + C17:0 + C18:0 + C20:0 + C21:0 + C22:0 + C23:0 + C24:0. MUFA = C14:1 + C15:1 + C16:1 + C17:1 + C18:1n9t + C18:1n9c + C20:1 + C22:1n9 + C24:1. PUFA = C18:2n6t + C18:2n6c + C18:3n3 + C18:3n6 + C20:2 + C20:3n6 + C20:4n6 + C20:3n3 + C22:5n3 + C22:2n6 + C22:6. Data are presented as mean ± SEM (*n* = 3) (NS—not significant, *p* > 0.05, * *p* < 0.05, ** *p* < 0.01, *** *p* < 0.001).

**Table 3:** The qRT-PCR primer sequences and annealing temperature.

Gene	Primer Sequences (5′→3′)	TM (°C)
*β-actin*	F: GTGACGTTGACATCCGTAAAGA	60
	R: GCCGGACTCATCGTACTCC	
*U6*	F: CTCGCTTCGGCAGCACA	60
	R: AACGCTTCACGAATTTGCGT	
*C/EBPα*	F: GCGGGAACGCAACAACATC	62
	R: GTCACTGGTCAACTCCAGCAC	
*CDK4*	F: GTCAGTTTCTAAGCGGCCTG	60
	R: CACGGGTGTTGCGTATGTAG	
*FABP4*	F: AAGGTGAAGAGCATCATAACCCT	52
	R: TCACGCCTTTCATAACACATTCC	
*SCD-1*	F: TTCTTGCGATACACTCTGGTGC	63.3
	R: CGGGATTGAATGTTCTTGTCGT	
*ACADL*	F: TGCCCTATATTGCGAATTACGG	63.3
	R: CTATGGCACCGATACACTTGC	
*ACOX2*	F: CATCCAACGTGACCCAGTGTT	63.3
	R: AAATGCGTTCAGGACCGTCTT	
*CDK2*	F: GCGACCTCCTCCCAATATCG	60
	R: GTCTGATCTCTTTCCCCAACTCT	
*LPL*	F: GGTTGCGCGTAGAGAGGATG	59.6
	R: CTCACGCTCTGACATGCCTTC	
*PPARα*	F: TACTGCCGTTTTCACAAGTGC	62
	R: AGGTCGTGTTCACAGGTAAGA	
*Adipor2*	F: TGTTCCTCTTAATCCTGCCCA	52.8
	R: CCAACCTGCACAAGTTCCCTT	
*FAS*	F: AGGTGGACTGGATACACAGAC	62
	R: TCTCCTGCCCAAACTCTTTGC	
*Cyclin E1*	F: CTCCGACCTTTCAGTCCGC	60
	R: CACAGTCTTGTCAATCTTGGCA	
*p21*	F: GATGGCTTCGACACCATTCC	60
	R: AGACGACACAGGTGAGGAAG	
*Cyclin D1*	F: CTCCGTATCTTACTTCAAGTGCG	60
	R: CTTCTCGGCAGTCAAGGGAA	
*p53*	F: CTCTCCCCCGCAAAAGAAAAA	60
	R: CGGAACATCTCGAAGCGTTTA	
*mmu-miR-126b-5p*	ATTATTACTCACGGTACGAGTT	60

## Data Availability

The data presented in this study is available in the article.

## References

[1] Singh G.M., Danaei G., Farzadfar F., Stevens G.A., Woodward M., Wormser D., Kaptoge S., Whitlock G., Qiao Q., Lewington S. (2013). The Age-Specific Quantitative Effects of Metabolic Risk Factors on Cardiovascular Diseases and Diabetes: A Pooled Analysis. PLoS ONE.

[2] Gregor M.F., Hotamisligil G.S. (2011). Inflammatory mechanisms in obesity. Annu. Rev. Immunol..

[3] Malik V.S., Popkin B.M., Bray G.A., Després J.-P., Hu F.B. (2010). Sugar-sweetened beverages, obesity, type 2 diabetes mellitus, and cardiovascular disease risk. Circulation.

[4] Pereira S.S., Alvarez-Leite J.I. (2014). Low-grade inflammation, obesity, and diabetes. Curr. Obes. Rep..

[5] He Y., Pan A., Wang Y., Yang Y., Xu J., Zhang Y., Liu D., Wang Q., Shen H., Zhang Y. (2017). Prevalence of overweight and obesity in 15.8 million men aged 15–49 years in rural China from 2010 to 2014. Sci. Rep..

[6] Meisinger C., Ezzati M., Di Cesare M. (2016). Trends in adult body-mass index in 200 countries from 1975 to 2014: A pooled analysis of 1698 population-based measurement studies with 19.2 million participants. Lancet.

[7] Zhang P., Du J., Wang L., Niu L., Zhao Y., Tang G., Jiang Y., Shuai S., Bai L., Li X. (2018). MicroRNA-143a-3p modulates preadipocyte proliferation and differentiation by targeting MAPK7. Biomed. Pharmacother..

[8] Fan Y., Gan M., Tan Y., Chen L., Shen L., Niu L., Liu Y., Tang G., Jiang Y., Li X. (2019). Mir-152 regulates 3T3-L1 preadipocyte proliferation and differentiation. Molecules.

[9] Peng Y., Chen F.-F., Ge J., Zhu J.-Y., Shi X.-E., Li X., Yu T.-Y., Chu G.-Y., Yang G.-S. (2016). miR-429 inhibits differentiation and promotes proliferation in porcine preadipocytes. Int. J. Mol. Sci..

[10] Li M., Wu H., Luo Z., Xia Y., Guan J., Wang T., Gu Y., Chen L., Zhang K., Ma J. (2012). An atlas of DNA methylomes in porcine adipose and muscle tissues. Nat. Commun..

[11] Sun Z., Ou C., Liu J., Chen C., Zhou Q., Yang S., Li G., Wang G., Song J., Li Z. (2019). YAP1-induced MALAT1 promotes epithelial–mesenchymal transition and angiogenesis by sponging miR-126-5p in colorectal cancer. Oncogene.

[12] Schober A., Nazari-Jahantigh M., Wei Y., Bidzhekov K., Gremse F., Grommes J., Megens R.T., Heyll K., Noels H., Hristov M. (2014). MicroRNA-126-5p promotes endothelial proliferation and limits atherosclerosis by suppressing Dlk1. Nat. Med..

[13] Xue S., Liu D., Zhu W., Su Z., Zhang L., Zhou C., Li P. (2019). Circulating MiR-17-5p, MiR-126-5p and MiR-145-3p are novel biomarkers for diagnosis of acute myocardial infarction. Front. Physiol..

[14] Dehghani M., Zarch S.M.A., Mehrjardi M.Y.V., Nazari M., Babakhanzadeh E., Ghadimi H., Zeinali F., Talebi M. (2020). Evaluation of miR-181b and miR-126-5p expression levels in T2DM patients compared to healthy individuals: Relationship with NF-κB gene expression. Endocrinol. Diabetes Nutr..

[15] Dashty M. (2013). A quick look at biochemistry: Carbohydrate metabolism. Clin. Biochem..

[16] Longo M., Zatterale F., Naderi J., Parrillo L., Formisano P., Raciti G.A., Beguinot F., Miele C. (2019). Adipose tissue dysfunction as determinant of obesity-associated metabolic complications. Int. J. Mol. Sci..

[17] Caruso J.A., Duong M.T., Carey J.P., Hunt K.K., Keyomarsi K. (2018). Low-molecular-weight cyclin E in human cancer: Cellular consequences and opportunities for targeted therapies. Cancer Res..

[18] Xiong Y., Hannon G.J., Zhang H., Casso D., Kobayashi R., Beach D. (1993). p21 is a universal inhibitor of cyclin kinases. Nature.

[19] El-Deiry W.S., Tokino T., Velculescu V.E., Levy D.B., Parsons R., Trent J.M., Lin D., Mercer W.E., Kinzler K.W., Vogelstein B. (1993). WAF1, a potential mediator of p53 tumor suppression. Cell.

[20] Dulić V., Kaufmann W.K., Wilson S.J., Tisty T.D., Lees E., Harper J.W., Elledge S.J., Reed S.I. (1994). p53-dependent inhibition of cyclin-dependent kinase activities in human fibroblasts during radiation-induced G1 arrest. Cell.

[21] Christy R., Yang V., Ntambi J., Geiman D., Landschulz W., Friedman A., Nakabeppu Y., Kelly T., Lane M. (1989). Differentiation-induced gene expression in 3T3-L1 preadipocytes: CCAAT/enhancer binding protein interacts with and activates the promoters of two adipocyte-specific genes. Genes Dev..

[22] Berger J., Moller D.E. (2002). The mechanisms of action of PPARs. Annu. Rev. Med..

[23] Ma Y., Liu D. (2013). Hydrodynamic delivery of adiponectin and adiponectin receptor 2 gene blocks high-fat diet-induced obesity and insulin resistance. Gene Ther..

[24] Tomita K., Oike Y., Teratani T., Taguchi T., Noguchi M., Suzuki T., Mizutani A., Yokoyama H., Irie R., Sumimoto H. (2008). Hepatic AdipoR2 signaling plays a protective role against progression of nonalcoholic steatohepatitis in mice. Hepatology.

[25] Liu Y., Michael M.D., Kash S., Bensch W.R., Monia B.P., Murray S.F., Otto K.A., Syed S.K., Bhanot S., Sloop K.W. (2007). Deficiency of adiponectin receptor 2 reduces diet-induced insulin resistance but promotes type 2 diabetes. Endocrinology.

[26] Kraus M., Greither T., Wenzel C., Bräuer-Hartmann D., Wabitsch M., Behre H.M. (2015). Inhibition of adipogenic differentiation of human SGBS preadipocytes by androgen-regulated microRNA miR-375. Mol. Cell. Endocrinol..

[27] Chegary M., te Brinke H., Ruiter J.P., Wijburg F.A., Stoll M.S., Minkler P.E., van Weeghel M., Schulz H., Hoppel C.L., Wanders R.J. (2009). Mitochondrial long chain fatty acid β-oxidation in man and mouse. Biochim. Biophys. Acta.

[28] Zhang D., Liu Z.-X., Choi C.S., Tian L., Kibbey R., Dong J., Cline G.W., Wood P.A., Shulman G.I. (2007). Mitochondrial dysfunction due to long-chain Acyl-CoA dehydrogenase deficiency causes hepatic steatosis and hepatic insulin resistance. Proc. Natl. Acad. Sci. USA.

[29] Bakermans A.J., Geraedts T.R., van Weeghel M., Denis S., João Ferraz M., Aerts J.M., Aten J., Nicolay K., Houten S.M., Prompers J.J. (2011). Fasting-induced myocardial lipid accumulation in long-chain acyl-CoA dehydrogenase knockout mice is accompanied by impaired left ventricular function. Circ. Cardiovasc. Imaging.

[30] Cox K.B., Liu J., Tian L., Barnes S., Yang Q., Wood P.A. (2009). Cardiac hypertrophy in mice with long-chain acyl-CoA dehydrogenase or very long-chain acyl-CoA dehydrogenase deficiency. Lab. Investig..

[31] Kimura I., Ichimura A., Ohue-Kitano R., Igarashi M. (2020). Free fatty acid receptors in health and disease. Physiol. Rev..

[32] Cohen P., Ntambi J.M., Friedman J.M. (2003). Stearoyl-CoA desaturase-1 and the metabolic syndrome. Curr. Drug Targets Immune Endocr. Metabol. Disord..

[33] Matsuzaka T., Shimano H. (2009). Elovl6: A new player in fatty acid metabolism and insulin sensitivity. J. Mol. Med..

[34] Du J., Shen L., Tan Z., Zhang P., Zhao X., Xu Y., Yang Q., Ma J., Jiang A.A., Tang G. (2018). Betaine supplementation enhances lipid metabolism and improves insulin resistance in mice fed a high-fat diet. Nutrients.

[35] Sang L.-J., Ju H.-Q., Liu G.-P., Tian T., Ma G.-L., Lu Y.-X., Liu Z.-X., Pan R.-L., Li R.-H., Piao H.-l. (2018). LncRNA CamK-A regulates Ca2+-signaling-mediated tumor microenvironment remodeling. Mol. Cell..

[36] Hengge U.R., Walker P.S., Vogel J.C. (1996). Expression of naked DNA in human, pig, and mouse skin. J. Clin. Investig..

[37] Bai H., Lester G.M.S., Petishnok L.C., Dean D.A. (2017). Cytoplasmic transport and nuclear import of plasmid DNA. Biosci. Rep..

